# Modular toolkit to facilitate molecular manipulations in mycobacteria

**DOI:** 10.1128/jb.00474-25

**Published:** 2026-03-16

**Authors:** Asis Kumar Khuntia, Ankita Dabla, Yogita Kapoor, Amit Chakraborty, Praveen Ebenezer Arputharaj, Vinay Kumar Nandicoori

**Affiliations:** 1 CSIR-Centre for Cellular and Molecular Biology 28569 , Hyderabad, India; 2 Academy of Scientific and Innovative Research (AcSIR) 550336, Ghaziabad, India; 3 BRIC-National Institute of Immunology 678582, New Delhi, India; Indian Institute of Technology Bombay, Mumbai, Maharashtra, India

**Keywords:** mycobacteria, gene expression, epitope tags, fluorescent reporters, integrative loci

## Abstract

Gene expression in mycobacteria relies on both integrative and episomal *Escherichia coli*-mycobacterium shuttle vectors. However, existing vectors have limited flexibility for adding epitope tags, selectable markers, and fluorescent reporters. To overcome this, we created a modular toolkit consisting of 32 integrative and eight episomal *E. coli*-mycobacterium shuttle vectors. Each vector features N- or C-terminal tags: SBP-HA-S, 3×-FLAG, GFP, and mCherry. The integrative vectors contain four different phage systems (L5, Giles, MS6, and Tweety), each with a distinct antibiotic resistance marker, enabling stable integration at various chromosomal sites. We validated the system in *Mycobacterium smegmatis* by expressing RbpA, an RNA polymerase-binding protein. Fluorescent tags confirmed Wag31’s polar localization, whereas co-expression with FtsZ illustrated their distinct localizations. The system’s flexibility was further shown by co-expressing four *Mycobacterium tuberculosis* sigma factors (SigA, SigC, SigG, and SigH) simultaneously. Additionally, an SBP-HA-S tandem affinity tag facilitated efficient two-step purification of RbpA with RNA polymerase and related regulators, reducing background compared to FLAG pull-down. This toolkit provides a versatile, reliable platform for gene complementation, protein localization, multi-protein co-expression, and native complex purification, supporting advanced genetic and cell biological studies in mycobacteria.

**IMPORTANCE:**

Molecular manipulation of pathogens is crucial for studying gene functions. Here, we have used existing knowledge and tools to create a new set of shuttle vectors with an expanded range of tags at both the N- and C-termini, along with antibiotics. This expansion allowed us to target four known integrative sites in mycobacteria and one episomal vector, resulting in the creation of 40 novel constructs. We have developed constructs that enable the simultaneous visualization of mCherry and GFP for co-localization studies. Importantly, for the first time in mycobacteria, we utilized the SBP-HA-S tandem affinity tag, which enables the retention of native protein complexes for pulldown studies. The tools generated in this study will be handy for mycobacterial research.

## INTRODUCTION

*Mycobacterium tuberculosis* (*Mtb*) remains the deadliest bacterial pathogen, responsible for approximately 1.25 million deaths each year worldwide (1). Its genome, approximately 4.4 Mb in size, encodes around 4,000 open reading frames (ORFs) that contribute to its complex physiology, including a thick, lipid-rich cell wall and the ability to persist in hostile host environments (2, 3). However, 52% of the genes are annotated as having putative functions or conserved hypothetical genes and are not characterized in *Mtb* (4, 5). Studying the function of these genes requires robust genetic tools, which are essential for understanding the genetics, physiology, and virulence of *Mtb*. The discovery of extrachromosomal DNA in *Mycobacterium spp*. enabled the development of various genetic tools (6–8). The pAL5000 plasmid from *Mycobacterium fortuitum* became a foundational origin of replication for constructing *Escherichia coli-*mycobacterium shuttle vectors (9, 10). Subsequent vectors, such as pYUB12 (stable transformation), pNBVl (color-based selection), and pMV261 (constitutive expression), greatly facilitated functional studies (11–13).

The disadvantage of using episomal shuttle plasmids is that they have multiple extrachromosomal copies, which leads to high levels of expression and requires ongoing antibiotic selection to maintain them. To address these issues, site-specific recombination (SSR) systems utilizing mycobacteriophage-derived attP/*int* elements have been used to insert genes at specific chromosomal loci (14–16). Early SSR-based vectors, derived from phage L5, allowed stable integration in both fast- and slow-growing mycobacteria (16). Later vectors broadened the toolset by incorporating integrases and *attP* sites from additional phages such as Giles, Ms6, and Tweety (17–19). However, residual integrase activity can still cause excision, which can be prevented using Cre or Flp recombinase systems that remove the integrase gene after insertion (20, 21).

Gene expression in *E. coli-*mycobacterium shuttle vectors was driven by constitutive promoters like hsp60 and pfurA, enabling high-level expression in vectors such as pMV261 and pMFA (13, 22). Later, regulated promoters were developed, such as the acetamidase switch, the first inducible system reported for mycobacteria (23). An anhydrotetracycline (ATc) inducible system—Tet-ON and Tet-OFF—was developed to induce or suppress expression by adding ATc (24). Other systems available for mycobacteria include pristinamycin, theophylline, and nitrile inducible systems, which activate expression when their respective inducers are added (25–27). Shuttle vectors containing combined inducible systems, such as the TetR/Pip-off dual system and Pip-on/Tet-on systems, have also been developed for tighter regulation and use in mycobacteria (28). Despite these advances, current vectors lack features such as flexible epitope tagging and multiple selectable markers, limiting studies involving co-expression or protein co-localization.

Previously, we developed a series of shuttle episomal and integrative vectors with expanded multiple cloning sites and a convenient FLAG tag at the N-terminus (29). However, these vectors were limited to the L5 integration site, which restricted our ability to express multiple genes simultaneously from different loci. This concept of utilizing various integrative loci and Cre-Lox systems was previously developed and successfully applied (14). We asked whether we could combine features from different shuttle vectors to create a series of vectors that could carry various tags at the N- and C-termini, utilize all four available integrative systems, and use the Cre-Lox system to remove antibiotic and integrase genes from the vector.

Here, we report the development of a suite of 40 *E. coli-*mycobacterium shuttle vectors, including 8 episomal and 32 integrative plasmids based on the L5, Giles, Ms6, and Tweety phage integrase systems. Each integrative vector includes a unique antibiotic resistance marker for simultaneous selection. All vectors utilize a common P_myc1_tetO promoter and support expression of up to four genes, with modular N- and C-terminal tagging options (3×-FLAG, SBP-HA-S, GFP, and mCherry) to facilitate protein localization and functional studies. The design of vectors is such that we can increase their repertoire by using these modules in combinations and adding additional features such as TetR or Rev-TetR genes to make these vectors regulatable.

## RESULTS

### Generation of constitutive, integrative, and episomal vectors

**Fig 1:**
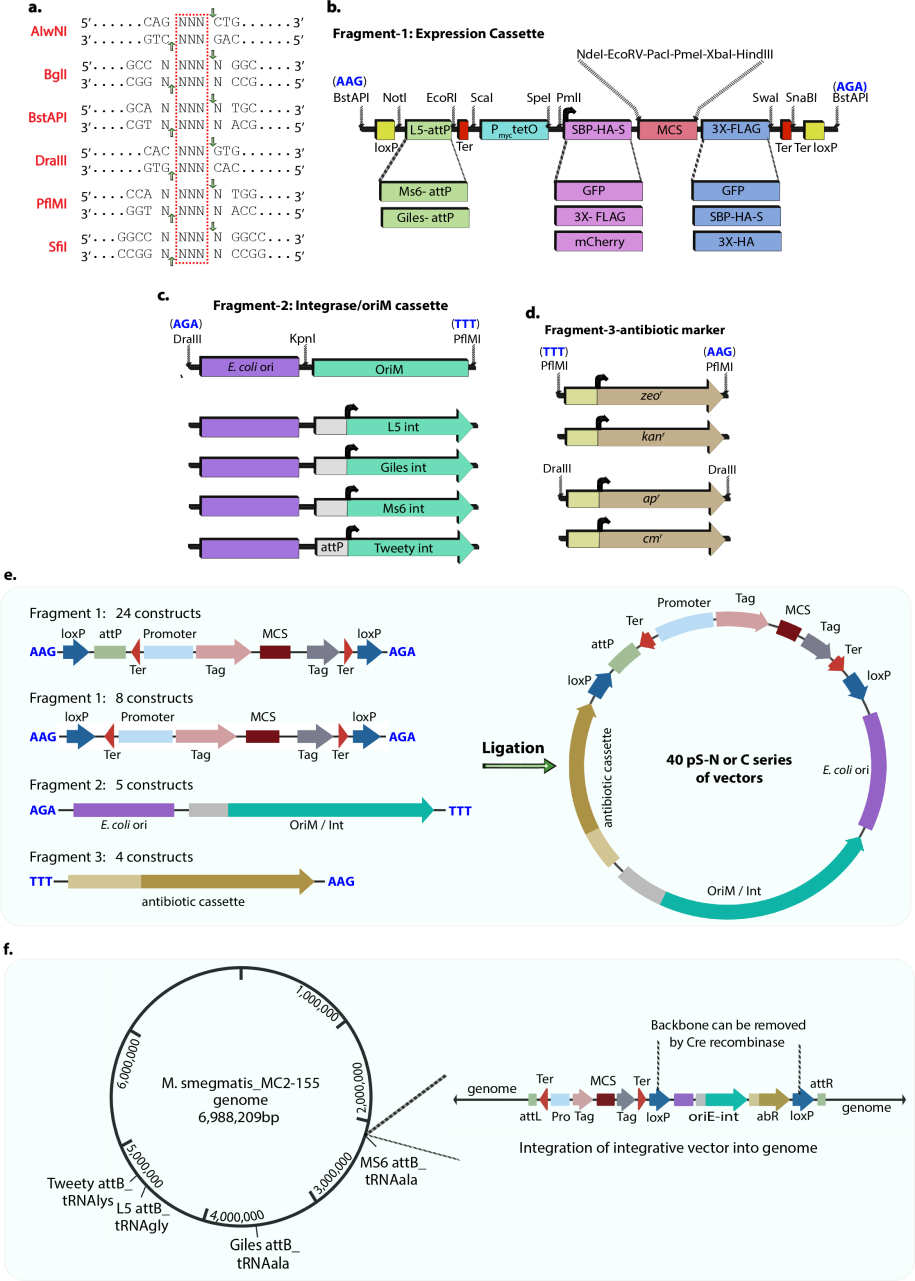
Overview of the *E. coli*–mycobacterium shuttle vector architecture. (**a**) List of selected Type IIP restriction enzymes and their discontinuous recognition sites. The central “NNN” indicates the cut site and the generation of compatible overhangs. (**b**) Schematic of the Fragment 1 (expression cassette), containing its respective components flanked with BstAPI (AAG, AGA) as 5’ overhangs at respective ends. (**c**) Fragment 2 was created by ligating *oriE* with integrase genes and oriM using the KpnI restriction site and DraIII flanking sites. Five variants of Fragment 2 were generated: four for integrative vectors and one for the episomal vector. (**d**) Fragment 3 contains four different antibiotic resistance genes, each providing a unique selectable marker. (**e**) A total of 40 unique integrative and episomal vectors by combining 32 Fragment 1 constructs, five Fragment 2 constructs, and four Fragment 3 constructs. (**f**) *M. smegmatis* genome map showing all four integration sites and the aftermath of integration. The region of the construct that can be removed with recombinase is indicated.

Multiple *E. coli*-mycobacterium shuttle vectors are available for cloning and expressing mycobacterial genes. However, the choice of N-terminal and C-terminal tags, as well as our ability to co-express multiple proteins simultaneously, has been a limiting factor. Here, we aimed to generate 32 different *E. coli*-mycobacterium shuttle vectors that can integrate at four distinct loci, as well as eight vectors capable of expressing genes episomally. To construct a functional shuttle vector, several DNA fragments must be assembled. These include (i) a gene expression cassette containing fusion tags, a multiple cloning site (MCS), a promoter, and a Shine-Dalgarno (SD) sequence for translation initiation (Fig. 1b); (ii) an origin of replication for maintenance in *E. coli*, along with either a mycobacterial origin of replication or an attP/integrase module for site-specific integration (Fig. 1c); and (iii) an antibiotic resistance marker for selection (Fig. 1d). The fragments were designed with compatible overhangs generated by restriction digestion, allowing efficient ligation into a complete shuttle vector—a strategy analogous to that used for constructing allelic exchange substrates (AES) in *Mtb* (30). Toward this, we used the flexibility provided by Type IIP restriction enzyme, which recognizes palindromic sequences separated by 3–5 nucleotides and cuts in such a way that generates three-nucleotide 3’ overhangs (Fig. 1a). Six commercially available enzymes fit the above requirement and hence were chosen for this study (Fig. 1a).

The expression cassette, Fragment 1, contains essential elements required for target gene expression (Fig. 1b). These include flanking loxP sites, necessary for Cre recombinase-mediated excision of the vector backbone that encompasses the antibiotic resistance cassette, the integrase gene, and *E. coli* oriE (20, 21). Additionally, it includes the L5 phage attachment site (attP), which can be substituted with alternative attP sites for Giles/MS6, enabling compatibility with their respective integrases. *E. coli* rrnB T1 transcriptional terminators and fd bacteriophage terminator were strategically positioned before the P_myc1_tetO promoter and after the multiple cloning site (MCS), respectively, to prevent unintended transcriptional read-through (24). The Pmyc1tetO promoter, which includes Tet operator (tetO) sequences, is followed by an optimal SD sequence, an N-terminal Streptavidin-Binding Peptide-Hemagglutinin-S protein (SBP-HA-S) tag for tandem affinity purification, an MCS containing NdeI, EcoRV, PacI, PmeI, XbaI, and HindIII sites, and a C-terminal 3×-FLAG tag for immunodetection and immunoprecipitation, all arranged sequentially in Fragment 1. Furthermore, SBP-HA-S, 3×-FLAG, green fluorescent protein (GFP), and red fluorescent protein (mCherry) tags were designed with compatible restriction enzyme sites to replace either N- or the C-terminal tags.

The other essential components of the *E. coli*-mycobacterium shuttle vector include an *E. coli* origin of replication (oriE) and an integrase gene for the integrative vectors, as well as a mycobacterial origin of replication (oriM) for episomal vectors. We fused oriE with the integrase genes for L5, Giles, MS6, and Tweety, or with oriM, using a unique KpnI site to create an oriE-integrase/oriM cassette, designated as Fragment 2 (Fig. 1c). For the Tweety phage, the integrase promoter is located within its attP region; hence, the entire attP-integrase cassette was amplified from ptb21-FtsZ-mCherry2B (19, 31). We designated the antibiotic selection cassette for apramycin (*apr^r^*), kanamycin (*kan^r^*), chloramphenicol (*cm^r^*), and zeocin (*zeo^r^*) as Fragment 3 (Fig. 1d). The existing L5 attP site in Fragment 1 was removed to generate both episomal and Tweety vectors.

**Fig 2:**
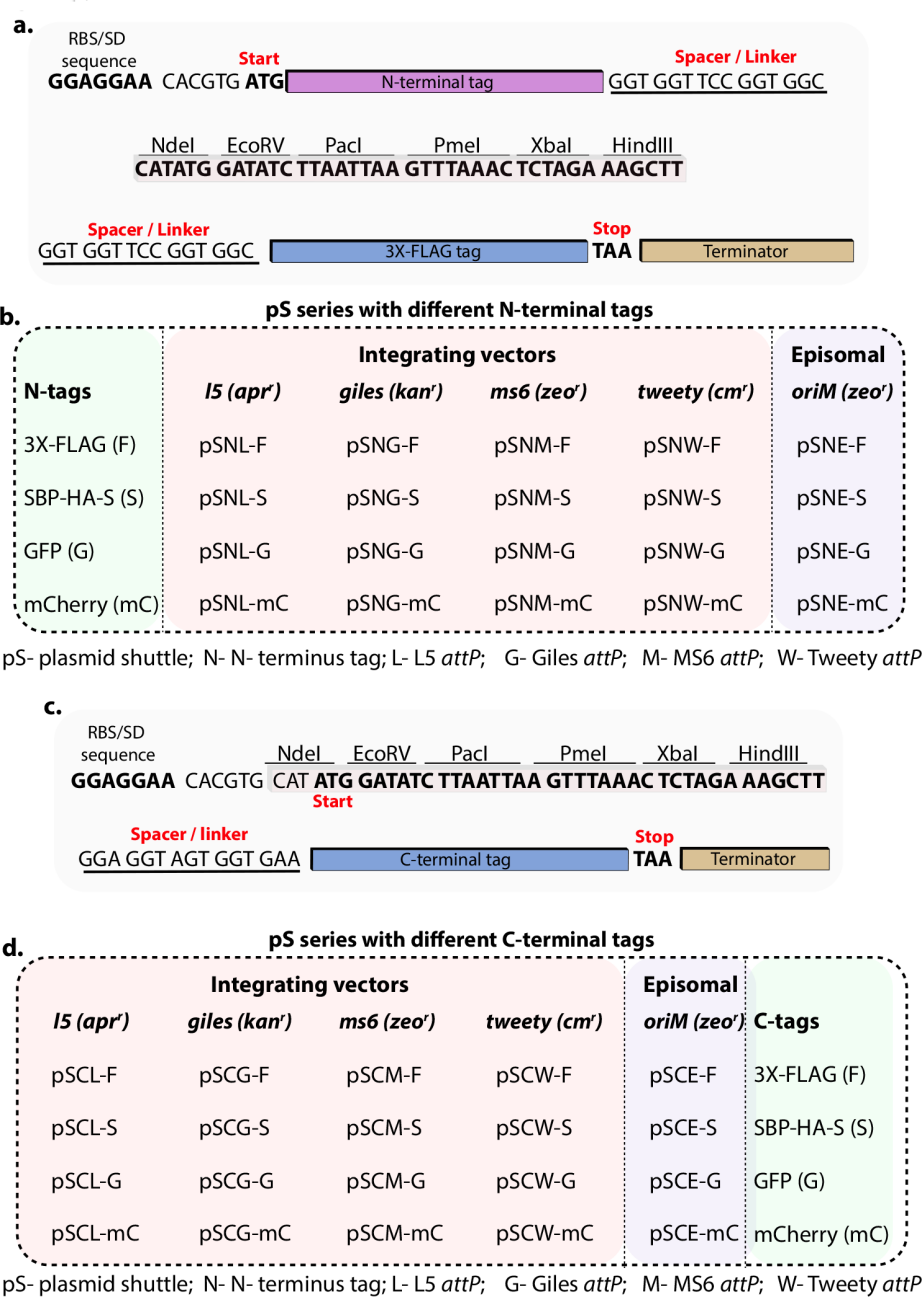
Construction of both N- and C-terminal-tagged vectors. (**a**) Schematic of the expression cassette modified to incorporate an N-terminal tag on the expressed protein. A flexible glycine-serine (GS) linker was inserted between the tag and the multiple cloning site (MCS). (**b**) Each of the five vector types was engineered with four different N-terminal tags—3×-FLAG, SBP-HA-S, GFP, and mCherry—resulting in the construction of 20 integrative and episomal vectors featuring distinct N-terminal tags. (**c**) Schematic showing the addition of the same four tags at the C-terminal end of the MCS, with a GS linker placed between the MCS and the tag. (**d**) A total of 20 vectors—comprising both episomal and integrative types—were constructed with C-terminal tags (3×-FLAG, SBP-HA-S, GFP, and mCherry) to enable flexible protein tagging.

In total, we generated 32 unique Fragment 1 inserts, with 8 inserts each for L5, Giles, MS6, and 8 for Tweety/episomal vectors. Additionally, five Fragment 2 inserts and four Fragment 3 inserts were created in the pENTR-D-TOPO vector for modular assembly (Fig. 1E). Fragments 1, 2, and 3 inserts in pENTR entry vectors were digested with suitable restriction enzymes and ligated in various combinations to produce final *E. coli*-mycobacterium shuttle vectors. The resulting integrative vectors target specific attB sites within the mycobacterial genome, each located in distinct tRNA genes, as shown in (Fig. 2f). Integration of the vector disrupts the corresponding tRNA gene. We did not include additional copies of the disrupted tRNA genes in these vectors because multiple copies of each respective tRNA gene are present in the genome, and disruption of a single copy does not affect the normal growth of either *Msm* or *Mtb*. In addition, the vector backbone, flanked by loxP sites, can be subsequently excised from the genome by Cre recombinase (Fig. 2f).

### Construction of unique N- and C-terminal-tagged vectors

Figure 2a is the schematic representation that depicts the features from the SD sequence to the terminator. Different N-terminal tags were placed upstream of the MCS and linked to it through a sequence encoding Gly-Gly-Ser-Gly-Gly (Fig. 2a). Similarly, the C-terminal tag was linked to the MCS via a spacer sequence encoding Gly-Gly-Ser-Gly-Gly (Fig. 2a). Sixteen of the 32 Fragment 1 inserts contained different N-terminal tags (3×-FLAG, SBP-HA-S, GFP, and mCherry) and the same C-terminal 3×-FLAG tag. Figure 2b shows the schematic of the features of the C-terminal-tagged vectors. None of the C-terminal-tagged vectors contained N-terminal tags. The MCS was connected to the C-terminal tags through the same linker sequence described above. Fragment 1 inserts related to C-terminal-tagged vectors included the same four tags (3×-FLAG, SBP-HA-S, GFP, and mCherry) at the C-terminal.

The nomenclature for the generated vectors is as follows: “p” denotes a plasmid, “S” indicates a shuttle vector, and “N” or “C” refers to vectors containing an N-terminal or C-terminal tag, respectively (Fig. 2c and d). The letters L, G, M, and W represent integrative vectors corresponding to the L5, Giles, MS6, and Tweety, respectively (Fig. 2c and d). The letter E represents episomal vectors containing OriM. The last letter stands for tags: F stands for 3×-FLAG, S for SBP-HA-S, G for GFP, and mC for mCherry. Antibiotic markers used for each integrative construct are shown in the table (Fig. 2c and d). Together, we constructed a total of 40 unique *E. coli*-mycobacterium shuttle vectors by modularly assembling Fragments 1, 2, and 3.

### Transformation efficiency of constructed shuttle vectors in *M. smegmatis*

**Fig 3:**
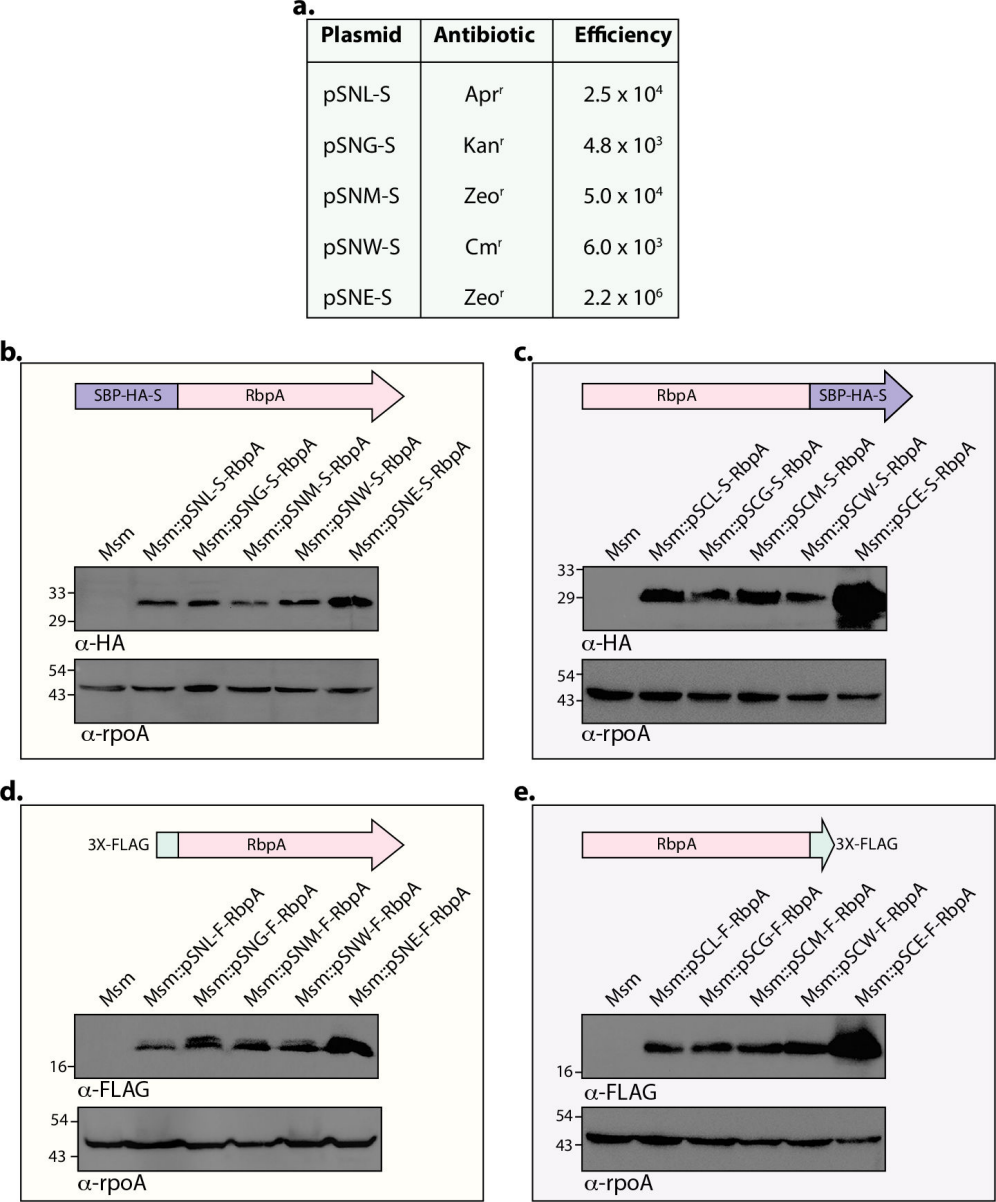
Expression of RbpA using N- and C-terminally tagged SBP-HA-S and 3×-FLAG vectors. (**a**) One microgram of each plasmid construct was electroporated into *Msm*. The number of resulting colonies was counted to assess transformation efficiency. (**b and c**) Western blot analysis of RbpA expression from N-terminally and C-terminally SBP-HA-S-tagged vectors, respectively, with the RNA polymerase alpha subunit (RpoA) used as a loading control. (**d and e**) Western blot analysis of RbpA expression from N- and C-terminal 3×-FLAG-tagged vectors, with RpoA used as the loading control.

Next, we set out to assess the transformation efficiency of the shuttle vectors. We evaluated the transformation efficiency of four integrating vectors, namely pSNL-S, pSNG-S, pSNM-S, and pSNW-S, and one episomal vector, pSNE-S. The remaining 35 vectors contain either one of the four integration sites with integrase or OriM; therefore, these five vectors serve as representative plasmids for the selected set. Transformation efficiency was evaluated by electroporating 1 μg of DNA into electrocompetent *Mycobacterium smegmatis* (*Msm*), a fast-growing saprophytic strain of mycobacteria. The number of colonies (CFU) obtained in a 1/100 dilution (10 ng) was used to determine efficiency. As expected, the episomal vector yielded the highest transformation efficiency (~2.2 × 10⁶ CFU/µg). The CFUs obtained with integrating vectors ranged from ~4 × 10³ to ~5 × 10⁴ CFU/µg (Fig. 3a). These vectors have been successfully employed in *M. tuberculosis*. Plasmid transformations were performed by electroporation using 1 μg of DNA. Although transformation efficiency was not formally quantified, we consistently observed more than 1,000 colonies per μg of DNA.

### SBP-HA-S and 3×-FLAG-tagged vectors robustly express proteins

To validate the vectors functionally, they were grouped into categories: those carrying 3×-FLAG or SBP-HA-S, either at the N or C-terminus, were classified as the first category (Fig. 3). Those with GFP or mCherry, either at the N or C-terminus, were classified as the second category (Fig. 5). The first category of vectors was validated by western blotting, while the second category of fluorescent-tagged vectors was examined using fluorescence microscopy. To validate the 20 vectors of the first category, the *rbpA* (MSMEG_3858) gene, which encodes the RNA polymerase-binding protein RbpA, a conserved and essential protein in actinomycetes, was used. RbpA is critical for the growth and survival of both *Msm* and *Mtb* and is a component of the transcription complex (32). The *rbpA* gene was PCR amplified using *Msm* genomic DNA as the template, and the amplicon was digested and cloned between the NdeI-HindIII sites.

The constructs were electroporated into the *Msm* strain, cultures were grown overnight, and the whole cell lysates (WCL) were prepared by bead beating. WCLs were resolved on 12% SDS-PAGE, transferred to a nitrocellulose membrane, and probed with either α-HA (for SBP-HA-S tag) or α-FLAG antibody (Fig. 3b and c). For validating vectors harboring C-terminal tags, the *rbpA* gene amplified without the stop codon was inserted into the NdeI-HindIII sites so that the C-terminal tags are in frame with the open reading frame of *rbpA* (Fig. 3d and e). The western blot data clearly show that both N- and C-terminal-tagged constructs strongly expressed RpbA. In some cases, two bands were observed in western blots when RpbA was tagged C-terminally with SBP-HA-S or N-terminally with 3×FLAG (Fig. 3c and d). The linker sequence used has been employed in multiple previous studies and is not known to contain protease cleavage sites; therefore, we believe that the appearance of multiple bands is more likely due to reduced tolerance of RbpA to N- or C-terminal tagging rather than proteolytic cleavage of the linker. As anticipated, the expression from the episomal constructs was higher compared with the integrative constructs because they are present in multiple copies.

### Excision of integrase and antibiotic marker (unmarking)

**Fig 4:**
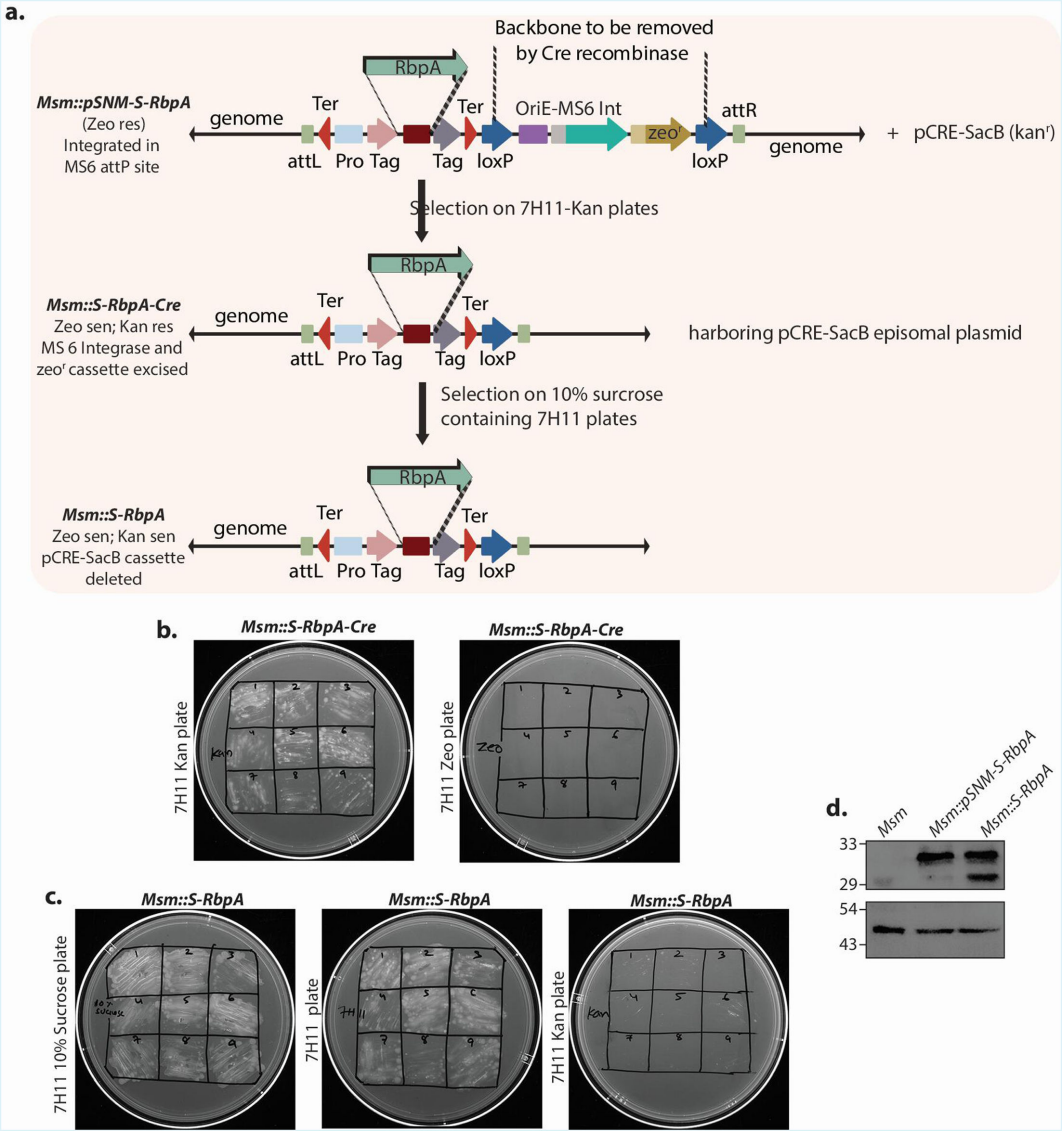
Excision of integrase and antibiotic marker. (**a**) The schematic shows the steps in the unmarking process. The top panel illustrates the genomic loci after integration of the pSNM-S-RbpA construct into the MS6 attB site in the *Msm::pSNM-S-RpbA* strain. This strain was electroporated with pCre-sacB, and recombinants were selected on Kan plates to generate *Msm::S-RpbA-Cre*. The pCre-sacB episomal plasmid was removed by plating the *Msm::S-RpbA-Cre* on 10% sucrose-containing 7H11 plates. (**b**) Nine independent colonies *of Msm::S-RpbA-Cre were* streaked onto 7H11-Kan and 7H11-Zeo plates. (**c**) Nine independent colonies *of Msm::S-RpbA were* streaked on 7H11-10% sucrose, 7H11, and 7H11-Kan plates. (**d**) RbpA expression was measured by western blot before and after backbone excision, showing similar levels in both cases.

Among the integrative vectors, MS6, Giles, and L5-derived vectors are designed so that the integrase and antibiotic cassettes can be excised (Fig. 1f and 4a) when Cre recombinase is expressed. To validate this, *the Msm-pSNM-S-RbpA* strain, originally Zeo^r^, was electroporated with pCre-SacB (Kan^r^) (33), and the transformants were selected on 7H9 Kan plates (Fig. 4a) to generate *Msm::S-RbpA-Cre*. Nine individual colonies were patched onto 7H9 Kan and 7H9 Zeo plates (Fig. 4b). While all grew on Kan plates, none grew on Zeo plates, indicating that the integrase and antibiotic marker backbone had been successfully excised. Subsequently, to remove the Cre recombinase from the *Msm::S-RbpA-Cre,* the strain was plated on 7H9 plates containing 10% sucrose, which, due to the presence of SacB in pCre-SacB, excises the integrated vector from the L5 integrative site (Fig. 4a) to generate the *Msm::S-RbpA* strain. Nine individual colonies were patched on 7H9 + 10% sucrose, 7H9, and 7H9 + Kan plates (Fig. 4c). While the colonies grew on 10% sucrose and plain 7H9 plates, they failed to grow on Kan plates, indicating that the Cre-SacB cassette has been removed from the L5 integration site. To confirm that we have not lost SBP-HA-S-RbpA integrated at MS6 loci, lysates prepared from *Msm*, *Msm-pSNM-S-RbpA,* and *Msm-S-RbpA* were probed with anti-RpoA and anti-HA antibodies (Fig. 4d). The expression of SBP-HA-S-RbpA could be detected in both *Msm-pSNM-S-RbpA* and *Msm-S-RbpA* strains, suggesting that we only excised the integrase + *Zeo* cassette, not the gene of interest (Fig. 4d). Since LoxP sites were the same in Giles and L5 integrative vectors, we believe that we will be able to unmark them too. We would like to point out that the tweety constructs cannot be unmarked, as the attP site is located outside the LoxP cassette. This is because the promoter for Tweety integrase overlaps with the attP site, and we could not segregate them while cloning.

### GFP- and mCherry-tags allow expression with a fluorescent tag

For the second category, vectors containing GFP or mCherry at either the N- or C-terminal tags are fused with Wag31. Wag31 is a scaffold protein in mycobacteria that includes a DivIVA domain, which is essential for survival and primarily localizes at the poles and partially at the septum. Wag31 is known to be important for cell division and elongation and has membrane tethering activity (34, 35). The Wag31 gene was amplified using specific primers, with or without its stop codon, and the amplicon was cloned into vectors with either N- or C-terminal GFP/mCherry. The constructs were electroporated into the *Msm* strain. While we were able to obtain colonies for the integrative constructs (16 out of 20), we did not get any colonies with the episomal constructs, namely pSNE-mG-wag31, pSNE-mC-wag31, pSCE-mG-wag31, and pSCE-mC-wag31. This is likely due to the constitutive overexpression of Wag31, which is probably toxic to the cell, preventing its survival.

**Fig 5:**
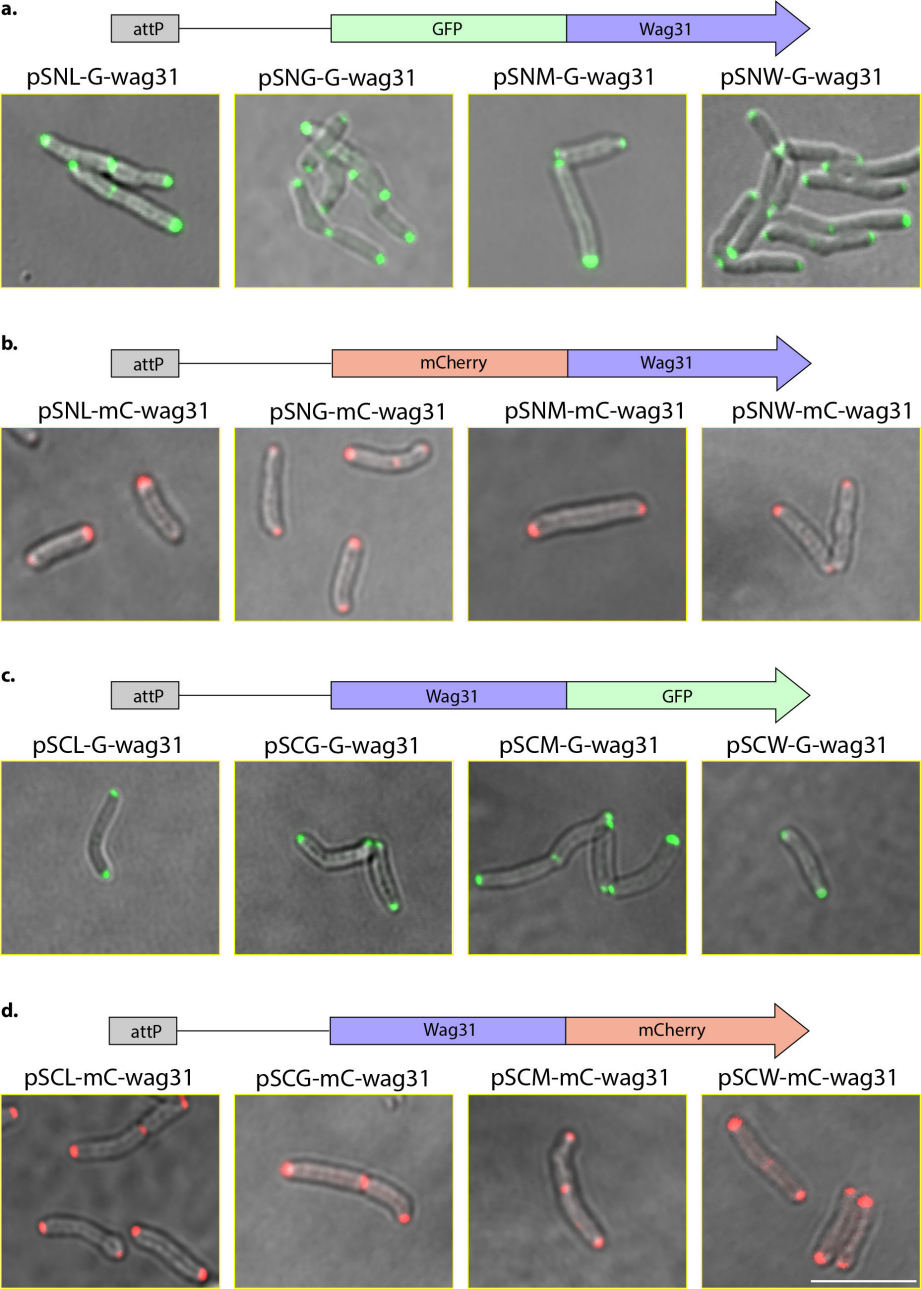
Localization of Wag31 in *Msm* using N- and C-Terminal GFP- and mCherry-tagged vectors. (**a and b**) Confocal microscopy images illustrating the expression and subcellular localization of Wag31 tagged with GFP or mCherry at the N-terminus. (**c and d**) Wag31 tagged similarly at the C-terminus, using four integrative vectors: L5, Giles, Ms6, and Tweety.

Colonies from four N-terminal GFP-tagged integrative vectors, each containing a distinct attP integration site, were analyzed using laser scanning confocal microscopy. Wag31 showed robust expression with a polar localization pattern consistent with previous findings (Fig. 5a) (35). Similar expression and localization were observed with N-terminal mCherry-tagged Wag31 (Fig. 5b). Vectors with C-terminal fluorescent tags were also validated. C-terminally tagged Wag31 with either GFP (Fig. 5c) or mCherry (Fig. 5d) displayed comparable expression levels and polar localization patterns, similar to those observed with N-terminal tagging. We also examined expression from empty vectors. The N-terminal GFP and mCherry vectors expressed the fluorescent proteins, whereas the C-terminal GFP and mCherry vectors did not, since C-terminal tag expression requires an in-frame open reading frame. Together, these results demonstrate that integrative fluorescent vectors tagged at either the N or C terminus are reliable tools for studying protein localization in mycobacteria.

### Simultaneous integration and co-expression of multiple genes

**Fig 6:**
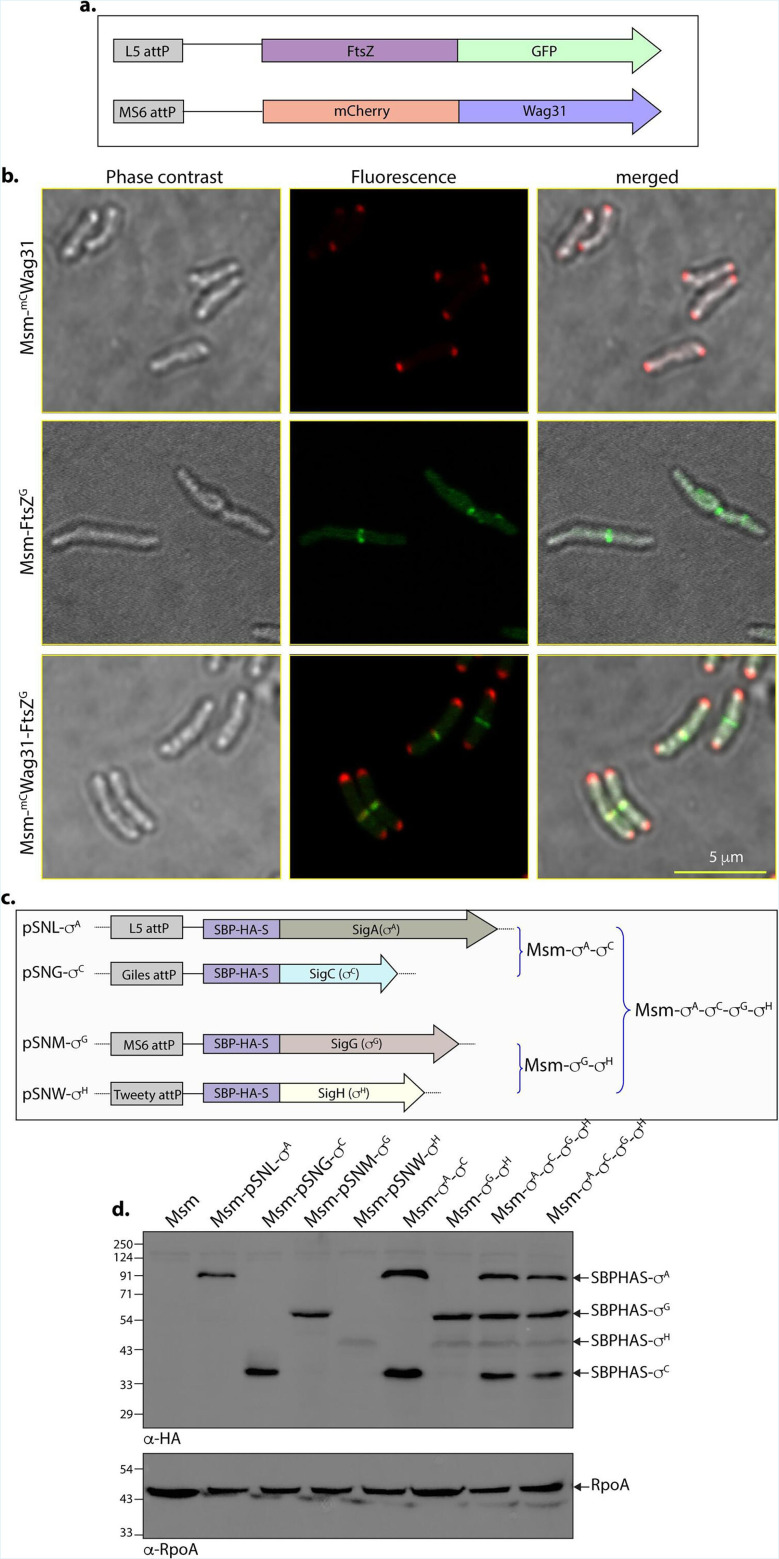
Gene co-localization and co-expression in *Msm* via constructed plasmid vectors. (**a**) Schematic representation of FtsZ-tagged with C-terminal GFP integrated at the L5 locus, and Wag31-tagged with N-terminal mCherry integrated at the Ms6 locus. (**b**) Confocal microscopy images showing the localization and co-localization of Wag31 and FtsZ. (**c**) Schematic depicting the four sigma factor genes (*sigA*, *sigC*, *sigG*, and *sigH*) and their respective integrative loci, along with the combinations used for co-expression studies. (**d**) Western blot analysis demonstrating expression of the four sigma factor genes from the L5, Giles, Ms6, and Tweety integration sites, respectively. Each gene was expressed individually, in pairs (SigA/SigC and SigG/SigH), and all four genes were co-expressed simultaneously within a single bacterial cell.

One of the main goals in developing these vectors is to enable the simultaneous co-expression of multiple proteins using different integration sites and antibiotic markers. Such simultaneous co-expression allows us to study protein interactions, co-localization, and create proteins with various tags. The different integrative vectors developed in this study have distinct antibiotic markers: L5 with the apramycin marker, Giles with the kanamycin marker, MS6 with the zeocin marker, and Tweety with the chloramphenicol marker. First, we aimed to determine if GFP and mCherry tags could be used for co-localization experiments. We selected two *Msm* cell division genes: Wag31, which localizes to the cell poles, and FtsZ, which localizes to the septum (34, 36). The *wag31* gene was fused to mCherry at the N-terminus (pSNM-mC), and *ftsZ* to GFP at the C-terminus (pSCL-G). These constructs were co-electroporated, and transformants were selected using zeocin and apramycin. Confocal microscopy confirmed proper expression and co-localization of mCherry-Wag31 and FtsZ-GFP at the poles and septum (Fig. 6a and b)

Subsequently, we tested the simultaneous expression of four *Mtb* sigma factor genes: *sigA*, *sigC*, *sigG*, and *sigH*. Each gene was cloned into a separate integrative vector (pSNL-S, pSNG-S, pSNM-S, or pSNW-S), each carrying an N-terminal SBP-HA-S tag. The constructs were electroporated individually and in pairs—pSNL-S-*sigA* with pSNG-S-*sigC*, or pSNM-S-*sigG* with pSNW-S-*sigH* (Fig. 6c)—and selected on plates containing apramycin and kanamycin or zeocin and chloramphenicol, respectively. This was followed by electroporation of the remaining two constructs, pSNM-S-*sigG* with pSNW-S-*sigH* into *Msm-s^A^-s^C^,* or pSNL-S-*sigA* with pSNG-S-*sigC* into *Msm-s^G^-s^H^*, and selecting them on plates containing zeocin and chloramphenicol or apramycin and kanamycin (Fig. 6c). We observed that the efficiency of co-transformation with these plasmids was approximately 1 log fold lower than that of single-plasmid transformations. When two plasmids were electroporated into strains already harboring two additional plasmids and selected on four antibiotics, the transformation efficiency decreased by approximately 2 log folds. Attempts to electroporate all four constructs simultaneously and select on plates containing four antibiotics were largely unsuccessful. Under this strong selection pressure, only one or two colonies were obtained, and none showed detectable simultaneous expression of all four constructs.

The cultures were grown in a growth medium that does not contain any antibiotics, and WCLs were prepared, with expression analyzed by western blot using α-HA antibodies. Western blot analysis confirmed the expression of SBP-HA-S-tagged sigma factors individually, in pairs, and the simultaneous expression of all four sigma factors (Fig. 6d). Notably, SigH showed consistently poor expression throughout the experiment. This may be due to sensitivity to vector context, reduced tolerance to tagging, or regulatory features of SigH, all of which require further investigation. Therefore, the vectors generated in the current study can be used for co-localization experiments and the simultaneous expression of multiple proteins.

### Tandem affinity purification (TAP) purification

Interactomes in mycobacteria are often performed with either 1× or 3×, FLAG, or HA tags. Using a single tag at either the N- or C-terminus for interactome studies, while effective, can lead to higher background noise. The SBP-HA-S tag contains three tags: SBP (streptavidin binding peptide) for binding to streptavidin; HA tag for detection by western blot; and S (s-peptide) tag for binding to S-protein beads. These systems, known as tandem affinity purification tags (TAP tags), are commonly used in eukaryotic systems to purify native complexes and identify native interactors with higher confidence (37, 38). In this study, we set out to evaluate the effectiveness of the SBP-HA-S tag for identifying interacting partners of RpbA. RbpA is essential for the growth and survival of both *Msm* and *Mtb* and is a part of the transcription complex. It interacts with the β subunit of RNA polymerase and with sigma factors SigA and SigB, helping to form a stable open promoter complex (32, 39–41). Although these interactions are well known, other interactors of RbpA still need to be identified. RbpA was chosen not only because of its biological importance but also as a candidate for purifying native transcription complexes and associated proteins with the SBP-HA-S tandem affinity purification tag.

**Fig 7:**
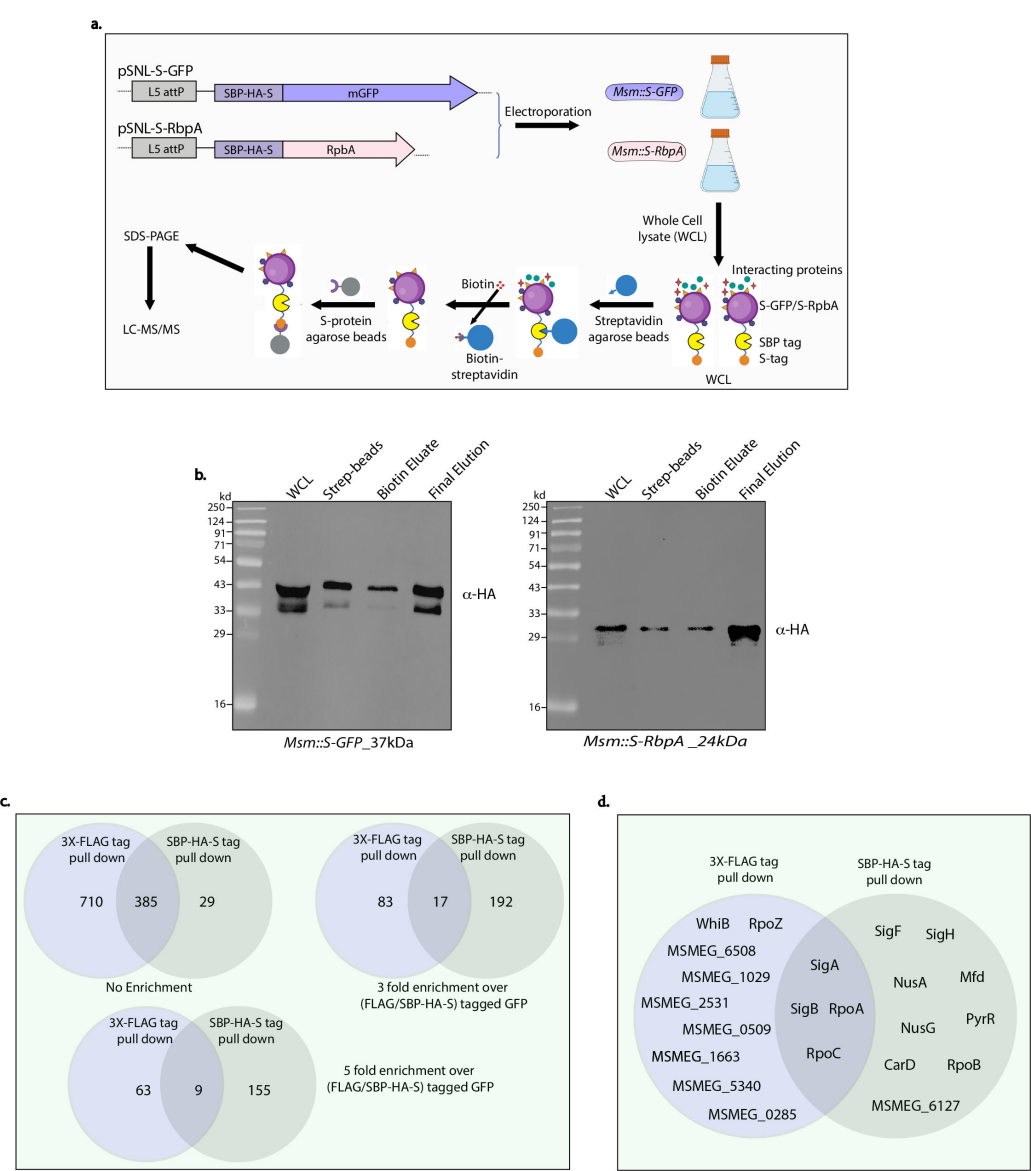
Tandem affinity purification (TAP) of RbpA and GFP, followed by liquid chromatography–tandem mass spectrometry (LC-MS/MS) analysis. (**a**) Diagram illustrating the workflow of the tandem affinity purification (TAP) process. RbpA was tagged at its N-terminus with an SBP-HA-S tag and subjected to TAP. The first affinity step used streptavidin agarose beads to capture the SBP tag, followed by a second affinity step using S-protein agarose beads to capture the S tag. A GFP-tagged SBP-HA-S protein served as a negative control. (**b**) Western blot analyses conducted at each purification stage—including bead retention, intermediate elutions, and final elution—confirm the presence of RbpA. (**c**) LC-MS/MS analysis identified RbpA-specific interacting proteins enriched through TAP purification compared to single-step purification with a 3×-FLAG tag. (**d**) RbpA is involved in transcription; therefore, we compared transcription-related interactors and found both shared and distinct proteins between the TAP and FLAG pull-downs. Notably, the TAP approach showed greater enrichment of transcription machinery components than the FLAG method.

The schematic (Fig. 7a) illustrates the purification process used for the SBP-HA-S-tagged GFP and RbpA proteins. In the first step, WCLs were incubated with streptavidin agarose beads. After thorough washing with phosphate-buffered saline (PBS) to remove unbound material, bound proteins were eluted with biotin. The eluate then underwent a second purification using S-protein agarose beads, with additional washes in PBS. The bound proteins were eluted by adding 2×-Laemmli buffer and heating the beads to 98°C for 5 min. The SBP-HA-S-tagged GFP construct served as a control to monitor nonspecific interactions. WCL, streptavidin-bound protein, biotin eluate, and final eluate were resolved on SDS-PAGE, transferred to nitrocellulose, and probed with anti-HA antibodies to assess the efficiency of each step and our ability to eluate bait proteins (Fig. 7b). Western blot analysis using α-HA antibodies (Fig. 7b) confirmed the presence of both GFP and RbpA during the purification process. To verify successful purification, proteins were collected at each step and analyzed by western blot, confirming the presence of the bait protein in both the bound and eluted fractions. Detection of both GFP and RbpA in the final eluates validated the samples for subsequent mass spectrometry analysis. Quantitative Liquid Chromatography-Tandem Mass Spectrometry (LC-MS/MS) analysis identified RbpA-associated proteins in both the TAP and FLAG pulldown samples. For comparison, we also performed a single-step pulldown using a 3×-FLAG-tagged RbpA and GFP construct, with 3×-FLAG-tagged GFP as the control.

Mass spectrometry analysis identified RbpA-associated proteins in both the SBP-HA-S and FLAG pulldown samples. We identified 1,095 proteins in FLAG-tagged (Table S4) and 414 proteins in SBP-HA-S-tagged (Table S5) RbpA purifications before subtracting proteins found in FLAG- and SBP-HA-S-tagged GFP. A total of 385 proteins were common between FLAG- and SBP-HA-S-tagged RbpA pull-downs. Initially, it appeared that there was a significantly higher background pulldown with FLAG compared to SBP-HA-S. Subsequently, we shortlisted only those proteins that were ≥3-fold or 5-fold enriched in FLAG- and SBP-HA-S-tagged RbpA pull-downs compared with FLAG- and SBP-HA-S-tagged GFP, respectively (Fig. 7c). With ≥ 3-fold enrichment, the total number of proteins identified in FLAG-RbpA pulldowns decreased to 100 from 1,095, and for SBP-HA-S-tagged RbpA, it decreased to 209 from 414. The number of common proteins also decreased from 385 to 17. Further increase ≥ 5-fold enrichment decreased the FLAG-RbpA pulldowns to 72 from 100, and for SBP-HA-S-tagged RbpA, it decreased to 164 from 209 (Fig. 7c). Together, our data suggest that FLAG pulldowns seem to have much higher background, and once the background is subtracted, the total numbers identified were 6.5% relative to those identified in the pulldown. In contrast, in the case of SBP-HA-S-tagged RbpA, despite subtracting the background, we retained 39.6% of the proteins that were originally identified.

We focused on transcription factors identified by both methods, given RbpA’s established role in transcription initiation. The overlapping interactors included RbpA and previously known RbpA interactors, such as SigA, SigB, and the β’ subunit (RpoC) of RNA polymerase. In the FLAG-tag pull-down approach, additional interactors comprised several transcription factors, including WhiB, MSMEG_6508, MSMEG_1029, MSMEG_2531, MSMEG_0509, MSMEG_1663, MSMEG_5340, MSMEG_0285, and the RNA polymerase ω subunit (RpoZ). Interestingly, the SBP-HA-S pull-down primarily identified components associated with the transcription complex and regulation (Fig. 7d). These included sigma factors SigF and SigH, elongation factors NusA and NusG, the RNA polymerase β subunit, the open complex-stabilizing factor CarD, the transcription-repair coupling factor Mfd, the anti-anti-sigma factor MSMEG_6127, and regulatory proteins such as PyrR, and a pyrimidine operon regulatory protein (Fig. 7d). Overall, the results suggest that the SBP-HA-S tag system offers a more specific, efficient, and reliable strategy for isolating protein complexes, such as the transcription machinery, compared to the single-step FLAG pull-down. The TAP method significantly reduces background contamination, thereby increasing confidence in the identification of biologically relevant RbpA interactors.

## DISCUSSION

Before the development of *E. coli*-mycobacterium shuttle vectors and the temperature-sensitive phagemid pHAE159 for mutant creation, molecular work in mycobacteria was limited (30). The discovery of the pAL5000 origin of replication, now used for episomal shuttle plasmids, along with the identification of L5 phage integrase and attP/attB sites, significantly advanced mycobacterial molecular techniques (16). Over the past two decades, additional phage integrases and their corresponding attP/attB sites have been identified, enabling integration at various loci. However, the ability to use multiple integration sites simultaneously remained unexplored. The Neiderweis lab developed the pML series of shuttle vectors, incorporating loxP sites, multiple integrases, and attP combinations (L5, MS6, and Giles) (14). Although these vectors permitted integration at different sites, the removal of the integrase and antibiotic marker required transforming with a construct expressing Cre recombinase before using another integrase and attP combination, due to the use of the same antibiotic marker.

Despite these innovations, some limitations remain regarding the versatility of multiple cloning sites, fusion tags, and antibiotic markers, which can affect the ability to express multiple genes simultaneously. In this report, we used four different integrase systems, a pAL5000-dependent episomal system, and four commonly used antibiotic markers for selecting mycobacteria. Additionally, we employed four different fusion tags at both the N and C-termini. We developed these modules in three separate units to successfully create 40 different *E. coli*-mycobacterium shuttle vectors. The modules are designed to be combined to produce shuttle vectors with various antibiotic markers (Fig. 1 and 2). The shuttle vectors with 3×-FLAG and SBP-HA-S tags developed in this study were confirmed by expressing *Msm* RbpA (Fig. 3), and successful unmarking of these vectors from the genome using Cre recombinase was confirmed (Fig. 4). The vectors tagged with GFP and mCherry were validated through Wag31 expression with these fluorescent markers. However, the episomal vectors with GFP and mCherry could not be confirmed since no colonies grew on the selection plates, probably because of the toxic effects caused by constitutive Wag31 overexpression in the bacteria (Fig. 5).

Co-expression, or the simultaneous expression of more than one protein, is crucial for various studies, such as validating protein interactions or demonstrating co-localization. Currently, the best examples of co-expression involve either one episomal and one integrative plasmid with different antibiotic markers or an integrative/episomal plasmid containing multiple promoters and MCS (29, 42–44). Two integrative loci were used simultaneously to investigate the localization of LamA1 with either FtsZ or Wag31, well-known markers for divisome and elongasome, respectively (45). pML vectors allowed simultaneous expression of three proteins in different integration loci; however, one had to remove the antibiotic marker after each integration event, as all of them harbored the same antibiotic marker (14). Here, we have used four integrative loci with four different antibiotics, which theoretically would allow us to transform them together and select on a plate containing all four antibiotics. However, this approach did not work, possibly because the bacteria were subjected to very stringent selection/stress due to the presence of four antibiotics. As a result, we tried with two integration constructs at a time (Fig. 6). We also could use two independent loci for localization experiments, indicating that the vectors developed can be used effectively for simultaneous expression of multiple proteins.

TAP tagging has been employed in mycobacterial shuttle vectors to isolate native complexes through a two-step purification process. The tags previously used included a combination of maltose-binding protein (MBP)-6×-His or STREP-6×His (46, 47). In eukaryotes, the SBP-HA-S tag was commonly used to identify interacting partners because of its high affinity and gentle biotin elution of SBP-tagged proteins from streptavidin beads, along with its strong binding to the S protein in the second stage (48–50). In this study, we examined the possible use of the SBP-HA-S tag for tandem affinity purification of RbpA, a transcription factor known to be strongly associated with RNA polymerase as part of the machinery. The results shown in Fig. 7 indicate that although we could identify most of the RNA polymerase subunits, sigma factors, and transcription elongation factors in SBP-HA-S pull-downs, they could not be identified when using 3×-FLAG-mediated immunoprecipitation.

The shuttle vectors developed in this study are expected to serve as valuable tools for researchers working in mycobacteriology. In addition to the 40 shuttle vectors reported here, we can create new vectors by combining different modules of Fragments 1, 2, and 3. Furthermore, modifying or synthesizing various fragments of Fragment 1 with constitutive and inducible promoters, along with their repressors, as well as controlling translation through theophylline riboswitches and incorporating other tags such as SBP-SBP, myc, and Strep, can broaden the range of available shuttle vectors. This approach should enable us to custom-generate shuttle vectors tailored for specific applications.

## MATERIALS AND METHODS

### Oligos, chemicals, enzymes, and antibodies

All oligonucleotides, chemicals, and antibiotics were sourced from Bioserve, Sigma-Aldrich, Merck, and Amresco. Restriction enzymes and the pENTR-D-TOPO (pENTR) cloning kit were obtained from New England Biolabs (NEB) and Thermo Fisher Scientific. Monoclonal antibodies targeting FLAG and HA epitopes were purchased from Sigma-Aldrich, while the polyclonal anti-RpoA antibody was produced in-house.

All oligonucleotides used in this study are listed in Table S1 of the supplemental material.

### Bacterial strains, growth conditions, and plasmids

*Mycobacterium smegmatis* mc^2^155 (*Msm*) was grown in liquid Middlebrook 7H9 medium (Difco) supplemented with 10% ADC (containing 5% bovine serum albumin fraction V, 2% dextrose, 0.85% NaCl, and catalase) at 37°C with shaking at 180 rpm. For solid media, Middlebrook 7H11 was supplemented with 10% OADC (0.06% oleic acid, 5% bovine serum albumin fraction V, 2% dextrose, 0.85% NaCl, and catalase). Antibiotic selection concentrations were as follows: kanamycin 50 μg/mL for *E. coli* and 25 μg/mL for *Msm*; Zeocin 25 μg/mL for *E. coli* and 50 μg/mL for *Msm*; chloramphenicol 50 μg/mL for *E. coli* and 55 μg/mL for *Msm*; and apramycin 30 μg/mL for *E. coli* and *Mtb* and 5 μg/mL for *Msm*.

All plasmids and bacterial strains used or generated in this study are listed in Tables S2 and S3 of the supplemental material.

### Generation of modules for assembly into an *E. coli*-mycobacterium shuttle vector

#### Fragment 1

Fragment 1, as shown in Fig. 1, was commercially synthesized and cloned into a pUC57 construct by GeneScript. The fragment was PCR amplified, and the amplicons were cloned into a pENTR-D-TOPO vector to generate pENT-F1_LNS_ (L: L5 *attP*, N: N-terminal, S: SBP-HA-S tag). While designing Fragment 1_LNS_, unique restriction enzyme sites were incorporated to enable replacement of various parts, such as the L5 *attP* site and the N- and C-terminal tags. To generate the Giles and MS6 versions of Fragment 1, the respective *attP* sites were amplified from pML1357 and pML1361 (14), and amplicons were digested with NotI-EcoRI and cloned into the corresponding sites in pENT-F1_LNS_ to generate pENT-F1_GNS_ (G: Giles *attP*) and pENT-F1_MNS_ (M: MS6 *attP*). For the Tweety/Episomal version, the L5 *attP* in pENT-F1_LNS_ was replaced with a double-stranded oligonucleotide harboring NotI and EcoRI overhangs to generate pENT-F1_NS_. Each of these variants was further modified to incorporate different combinations of N- and C-terminal tags (Table S2).

To generate constructs containing only the C-terminal 3×-FLAG tag, a double-stranded oligonucleotide with PmlI and NdeI sites was cloned into the corresponding sites in pENT-F1_NS_ constructs to replace the N-terminal SBP-HA-S tag with no tag to generate pENT-F1_CF_. The N-terminal SBP-HA-S tags were replaced with a double-stranded oligonucleotide with PmlI and NdeI to generate pENT-F1_LCF_ (C: C-terminal, F: 3×-FLAG tag) or pENT-F1_GCF_, or pENT-F1_MCF_ constructs. Subsequently, the C-terminal 3×-FLAG was replaced by GFP, mCherry, and SBP-HA-S tags were PCR amplified from pML1357 and ptb21-FtsZ-mCherry2B, and pENT-Frag1_LNS_ with primers harboring a glycine-serine linker (gly-gly-ser-gly-gly) at the N-terminus, and the amplicons were digested with HindIII-HpaI and cloned into the corresponding sites in pENT-F1_CF,_ pENT-F1_LCF_, pENT-F1_GCF_, or pENT-F1_MCF_ constructs to generate pENT-F1_C_-G/mC/S series of constructs (Table S2).

To generate various N-terminal tagged constructs, GFP, mCherry, and 3×-FLAG were amplified from pML1357 and ptb21-FtsZ-mCherry2B, and pENTR-Frag1_LNS_ with primers harboring a glycine-serine linker (gly-gly-ser-gly-gly) at the C-terminus, and the amplicons were digested with PmlI-NdeI and cloned into the corresponding sites in pENT-F1_NS_, pENT-F1_LNS_, pENTR-F1_GNS_ or pENTR-F1_MNS_ constructs to generate pENT-F1_N_-F/G/mC series of constructs (Table S2).

#### Fragment 2

To create Fragment 2 constructs, the *E. coli* origin of replication was amplified from pML1357 using a phosphorylated forward primer with a compatible DraIII site and a reverse primer with a KpnI site. OriM/Integrase was amplified from pNit-1, pMV361, pML1357, pML1361, and ptb21-FtsZ-mCherry2B, respectively (13, 14, 29, 31), with primers containing a KpnI site in the forward primer and a phosphorylated reverse primer with a compatible DraIII site. *E. coli oriE* and OriM/Integrase amplicons were digested with KpnI and ligated with pENT-F1_L_ digested with HpaI-EcoRV to generate pENT-oriE-oriM constructs (Table S2).

#### Fragment 3

Four antibiotic resistance genes were PCR amplified and cloned into pENTR-D-TOPO. The kanamycin resistance gene was derived from pVV16 (29), while the apramycin resistance gene was PCR amplified from pMV261-apra (a gift from Prof. Jacobs, W. R. The chloramphenicol resistance gene was PCR amplified from the pVR1 vector (51). The zeocin resistance gene was amplified from pGH1000A::zeo-ID (31). In total, 32 Fragment 1 modules, 5 Fragment 2 modules, and 4 Fragment 3 modules were generated for the construction of final shuttle vectors (Table S2).

All Fragment 1, 2, and 3 constructs made in this study are listed in Table S2 of the supplemental material.

### Transformation efficiency

For the determination of the transformation efficiency of newly constructed vectors, 1 µg of each plasmid type was electroporated into competent *Msm,* and colonies were counted after 3 days on 7H11 agar plates containing the appropriate antibiotics. Transformation efficiency was calculated as the number of colonies that appeared after transforming 1 µg of plasmid. Unmarking of mutants was performed using pCre-SacB-Kan (a gift from Adrie J. C. Steyn) as described (33).

### Lysate preparation and western blot analysis

Competent *M. smegmatis* cells were prepared using established protocols (34). For transformation, 150 μL of freshly prepared competent cells was electroporated with 500–1,000 ng of plasmid DNA. To evaluate constitutive protein expression, bacterial cultures were grown to mid-log phase (OD₆₀₀ ≈ 0.6), harvested, and resuspended in phosphate-buffered saline containing 5% glycerol (PBSG) and protease inhibitor phenylmethanesulfonyl fluoride (PMSF). Cell lysis was performed using a Mini-BeadBeater (BioSpec Products) with 0.1-mm zirconia beads. Lysates were clarified by centrifugation at 16,100 × *g* for 30 min at 4°C, and total protein concentration was determined using the TaKaRa BCA assay kit. For immunoblotting, 20–50 μg of total protein from clarified lysates was separated on 12% SDS-PAGE gels, transferred to nitrocellulose membranes, and processed using standard western blot protocols (34). Primary antibodies were used at the following dilutions: anti-RpoA (rabbit, 1:10,000), anti-HA (rabbit, Sigma, 1:10,000), and anti-FLAG (mouse, Sigma, 1:3,000).

### Confocal microscopy imaging

*Msm* strains harboring fluorescent constructs were grown to mid-log phase (0.4–0.6 OD_600_), then washed once with PBS containing 0.1% (vol/vol) Triton X-100. Cells were seeded onto molded 1% agarose pads prepared with 7H9 medium for imaging. Fluorescence was observed using an Olympus FV3000 confocal laser-scanning microscope equipped with a PLAPON60XOSC2 objective. GFP (488 nm) and RFP (561 nm) channels were used for imaging. For each strain, 3–5 images were acquired, and experiments were performed in biological duplicates.

### Tandem affinity purification of RbpA and LC-MS/MS

*Msm* strains *S-GFP* and *S-RbpA* were grown in 7H9-ADC medium until the OD_600_ reached approximately 0.6. Cultures were pelleted at 3,500 rpm for 10 min at 4°C, followed by bead beating in 1× PBSG for cell lysis. The lysates were passed through 0.2-μm filters to remove cellular debris. Four milligrams of cell lysate were incubated with 100 μL of streptavidin agarose beads overnight at 4°C. Beads were washed three times with 1× PBS and eluted using biotin (2 mg/mL) for 2 h at room temperature. The eluate was then incubated with 50 μL of S-protein agarose beads for 2 h at 4°C, followed by three additional washes with 1× PBS. Proteins were eluted from the S-protein beads by boiling in 2× Laemmli buffer.

For mass spectrometry analysis, the eluted proteins were separated by SDS-PAGE (12% gel) until the dye front migrated ~1.5 cm into the resolving gel. Immunoprecipitated samples were reduced using 5 mM tris (2-carboxyethyl)phosphine (TCEP), alkylated with 50 mM iodoacetamide, and then digested with 1 μg of trypsin for 16 h at 37°C. Quantitative liquid chromatography-tandem mass spectrometry (LC-MS/MS) identified peptides. Data analysis was performed using Proteome Discoverer (version 2.2.0.388), and peptide/protein identifications were matched to the *Mycobacterium smegmatis* database (UniProt reference: UP000000757). For stringent analysis, only proteins identified with at least two peptide-spectrum matches (PSMs) were considered. Peptide abundance ratios were calculated for each replicate using the GFP control as a reference. Proteins with an abundance ratio ≥10 in at least two out of three replicates were retained for further analysis. The distribution of proteins between FLAG and SBP-HA-S tags, both before and after applying the abundance ratio filter, was visualized using Venn diagrams generated with the InteractiveVenn web service (https://www.interactivenn.net/).

Mass spectrometry data files for RbpA tagged with FLAG and SBP-HA-S used in this study are provided in Tables S4 and S5 of the supplemental material.

## SUPPLEMENTAL MATERIAL

Tables S1 to S5 jb.00474-25-s0001.xlsx Primers, plasmids, strains, and mass spectrometry data.

ASM does not own the copyrights to Supplemental Material that may be linked to, or accessed through, an article. The authors have granted ASM a non-exclusive, world-wide license to publish the Supplemental Material files. Please contact the corresponding author directly for reuse.
